# Energy Sources in Neonatal Surgery: Principles and Practice

**Published:** 2014-04-01

**Authors:** Shandip K Sinha, Anjan Dhua

**Affiliations:** 1 Department of Pediatric Surgery, Maulana Azad Medical Collage and Lok Nayak Hospital New Delhi, India-110002; 2 Department of Pediatric Surgery, Pondicherry Institute of Medical Sciences, Pondicherry, India- 605014

Pediatric surgeons need precise and advanced energy sources for performing procedures on neonates. The technologies have also advanced and industry is continuously upgrading to develop solutions that perform precise and effective action and at the same time is safe and reliable to both the patient as well to the theatre personnels [1]. The major energy sources for neonatal surgery available are electrosurgery and its modifications, ultrasonic dissectors, lasers, and cryotherapy. Of these, electrosurgery and ultrasonic energy is frequently used. A neonatal surgeon must have a basic understanding of the instruments for its safe and optimal use. This article deals with the basics physics of the instruments and how common understanding and precautions can make them safe, both for neonates and surgeons.

**Electrosurgery**

Electrosurgical devices are indispensable in while performing neonatal surgery. It is important that the surgeon understands the underlying principles and modifies it appropriately for optimal use. Although technologies have evolved, complications can still occur on the surgical patient, especially when the patient is a neonate. A burn or a similar accident can produce a lifelong blemish or lead to loss of function of an organ or limb. 

**Principles of electrosurgery**

Electrosurgery has been described as utilizing high-frequency electrical current to create a desired surgical effect [2]. Effect of high frequency current on the patient is thermal and not electrical. The delivered current passes through and heats the tissues. [Table 1]

**Figure:**
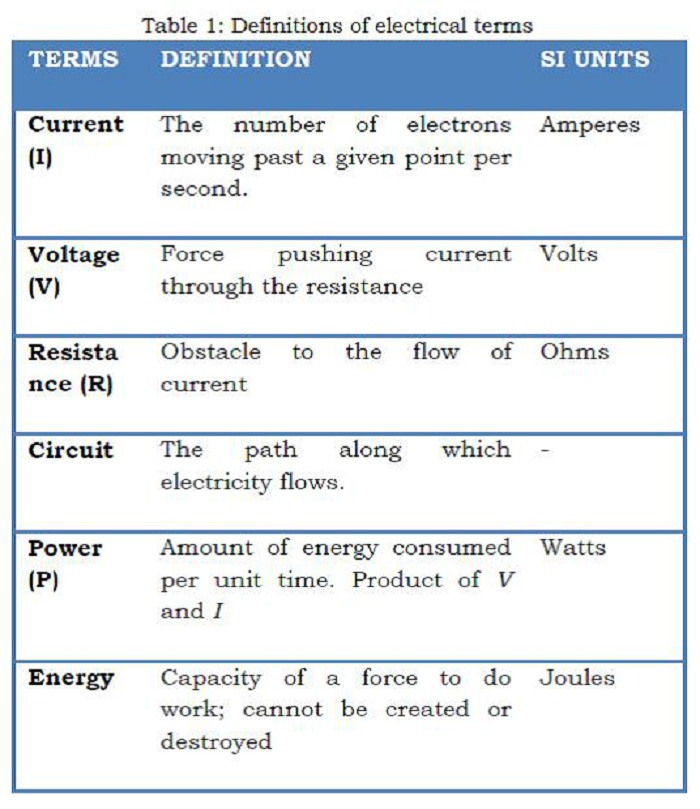
Table 1: Definitions of Electrical Terms

Because nerve and muscle stimulation cease at 100 kHz, electrosurgery can be performed safely at frequencies above 100 kHz. An electrosurgical generator takes 60 cycles current and increases the frequency to over 200-300 KHz per second (Fig. 1). Because the frequencies typically used for surgery are around 500 KHz, which is close to the frequency of, amplitude modification (AM) radiobroadcasts, the term “radiofrequency electro surgery” is also used [3]. The schematic arrangement of the various applications in the radiofrequency spectrum is shown in Fig. 2.

**Figure:**
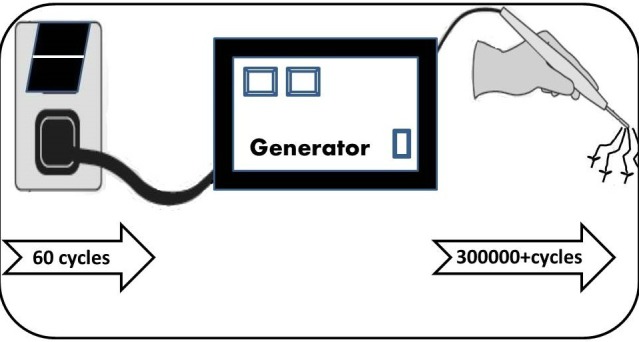
Figure 1: Schematic figure of the electrosurgical generator that converts the 50-60 cycles AC current to 300-500 K Hz frequency that produces various surgical effects at the hand piece.

**Figure:**
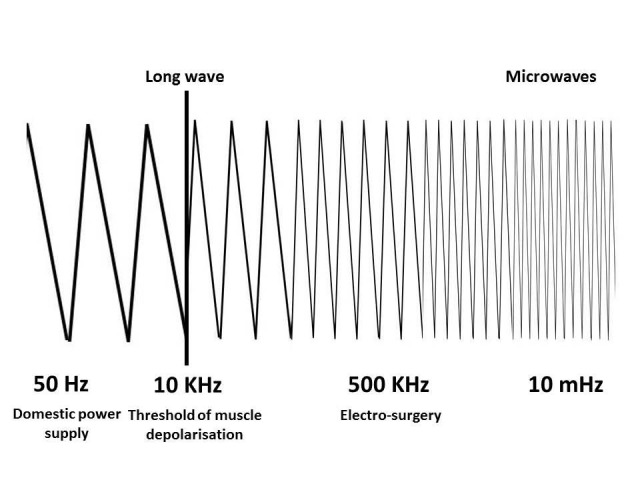
Figure 2: Schematic depiction of various applications of radiofrequency waves along the spectrum.

Electrical circuits and Ohm’s Law are two basic concepts upon which the electro surgery is based upon (Table 1). Electrical circuit is an uninterrupted pathway of flowing electrons and Ohm’s Law describes the actions of a given circuit:

Voltage = Current X Resistance

In electrosurgery, the generator provides voltage, and current is delivered to the tissues through the electrode tip of the instrument. Resistance to current is inherent with all human tissues. The electrical circuit is completed by patient return electrode, which offers a low-resistance pathway for current to return to the generator from the patient [4].

D’Arsonval discovered that the electricity could cause body temperature to rise. The temperature change was noted to be a function of the current density [5]. The transformation of electrical energy into heat occurs in accordance with Joules Law and can be expressed by the following formula:

Energy (heat generated) = (current/cross-sectional area) 2 X resistance X time

Heat given off is a function of current density (current per cross-sectional area), resistance and time. The amount of thermal energy delivered and temperature achieved will dictate the observed tissue effects (Table 2). If we modify the amount and time of thermal energy delivery, different results can be achieved. This is done by electrosurgical units (ESU). The current output of electrosurgical generators can be modulated both in terms of current density and time to deliver different waveforms to the tissue. 

 Tissue effects, which can be achieved with these generators, can be roughly divided into three basic groups: cutting, coagulation or desiccation and fulguration.

**Figure:**
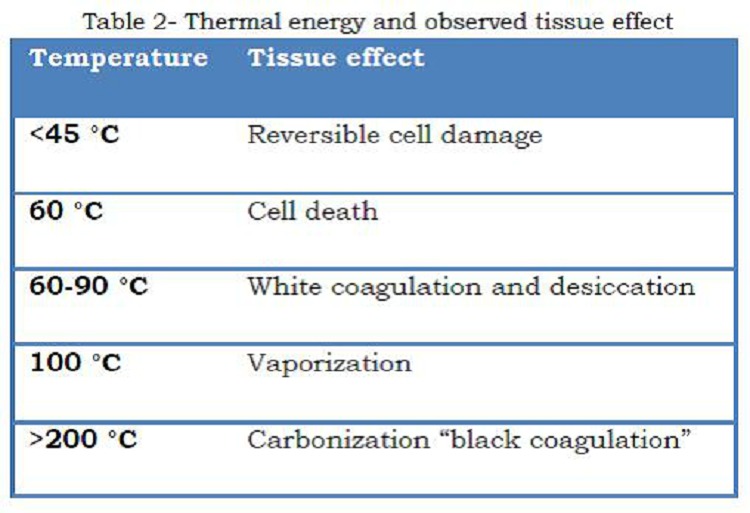
Table 2: Thermal energy and observed tissue effect

- Electrosurgical cutting divides tissue with electric sparks that direct intense heat (>100°C) to the tissue over a very limited surface area, producing maximum current density and delivering the greatest amount of heat over a very short time, leading to rapid expansion of the intracellular contents and explosive vaporization [8]. The output labeled “cut,” when set at “pure,” provides a continuous and relatively low-voltage waveform (Fig. 3a).
- Electrosurgical desiccation or coagulation - when tissue temperature is maintained at 60-95°C (by reducing current density leading to less heat production), protein coagulation and dehydration or desiccation occurs. “Coagulation” waveform is an interrupted, dampened, and relatively high-voltage waveform (Fig. 3b). Typically, the current is “on” only 6% of the time, referred to as a 6% duty cycle.
- Electrosurgical Fulguration is a process whereby the tissue is superficially coagulated by repeated [interrupted] high-voltage electrosurgical arcs that continue to elevate the temperature by resistive heating to beyond 100°, reaching levels of 200°C and more. In addition to coagulation and desiccation the high temperature results in carbonization (black carbonization).

**Figure:**
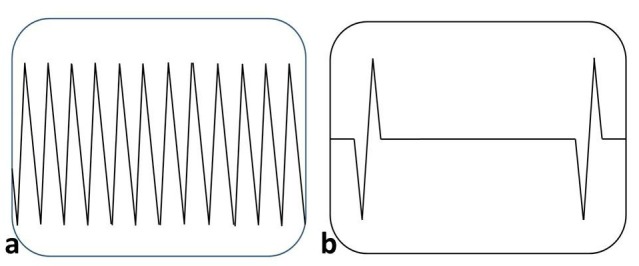
Figure 3a: showing the continuous waveform generated in ESU for pure “Cut” function, Figure 3b: interrupted waveform for a “Coagulation” function

While most ESUs have two outputs labeled cut and coagulation (or “coag”), these terms do not accurately reflect the appropriate tissue effect of the energy. Very high current density concentrated at the tip of an electrode can be used to heat and vaporize cells; slightly lower current density may desiccate and coagulate, while the same current spread over a large area may have no impact on the cells as seen at dispersive electrode (patient plate).

**Electrosurgical Generators (ESG)/ (ESU)**

There are two types of electrosurgical generators:

- Ground referenced generators (typically older, outdated units)
- Isolated generators (today’s state-of-art technology)

Ground referenced generators - The current passes through the patient and returns to the generator, which is linked to ground. The problem is the current can go to any grounded object other than the pad (ECG electrodes, operation table, metal objects) and cause alternate site burns. Ground referenced generators are now obsolete.

Isolated generators – These generators isolate the current from ground and do not allow significant current to seek alternate pathways to ground. The current must return through the dispersive pad to the generator.

**Techniques of delivery**

There are two basic types of electrical circuits: monopolar and bipolar.

Monopolar (mono terminal) is an electrosurgical technique in which the tissue effect takes place at a single active electrode and is dispersed (circuit completed) by a patient return electrode/ dispersive electrode (Fig. 4). It is important to remember that the dispersive electrode is just as capable of producing injury as the active electrode unless the patient return pad has large contact over conductive tissue to provide a low current density. There are now many inbuilt safety mechanism {return electrode monitoring (REM)/ contact quality monitor (CQM)} to prevent this complication [9]. The patients return plate pads are split and electrosurgical device through impedance measurement between the two (split pad) can sense the contact area. When extreme variations or very high/ low impedance appears, the CQM will lead to an alarm and can lead to deactivation of the output energy to prevent potential patient injury.

**Figure:**
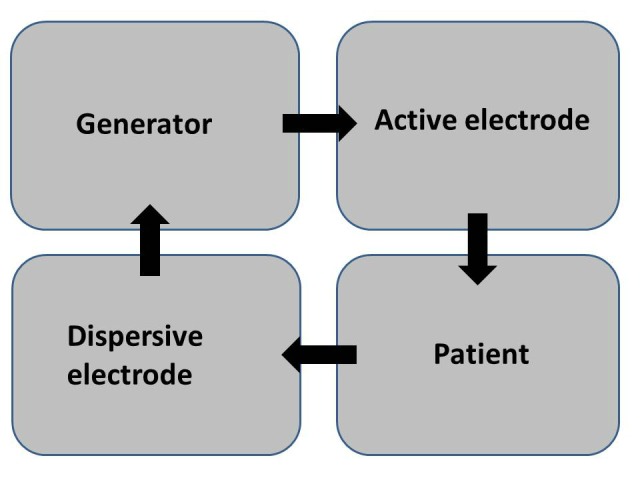
Figure 4: Circuit of a monopolar electrosurgical system

Most ESUs have two outputs labeled cut and coagulation (or “coag”). As explained earlier, these terms do not accurately reflect the appropriate tissue effect (cutting, coagulation or desiccation and fulguration) and use of the energy. Also there are no standards and the power and duty cycle can vary from one manufacturer to other. In addition a third modulated version popularly known as “blend” also exists. “Blended” outputs are interrupted (or modulated) versions of the continuous or pure “cut” waveforms. When the current is interrupted, and therefore reduced, while the wattage is held constant, the generator increases the voltage of the output (W = V × I). 

The surgeon by manipulating the ESU output, area of contact and distance of the electrode from the tissue can get desired appropriate tissue effect (cutting, coagulation or desiccation and fulguration) (Table 3). 

**Figure:**
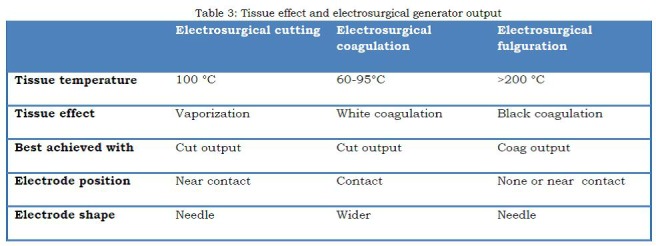
Table 3: Tissue effect and electrosurgical generator output

Argon beam - Electrosurgical delivery of energy using monopolar instruments can be enhanced by incorporating a stream of argon gas to improve the surgical effectiveness in maintaining hemostasis over larger surfaces. Traditional electrosurgical monopolar electrode does not function in a liquid (blood) environment because the current is dispersed. The argon beam unit overcomes this problem by adding a column of Argon gas passing over the electrode, in line with its tip. The argon gas becomes fully ionized by the electrosurgical energy and also acts to displace (blow away) the blood. Because Argon is a noble gas, it allows the current to arc from the electrode to the underlying tissue, following the path of the column of gas, creating a diffuse superficial coagulation ideal for obtaining hemostasis over large surface areas. The Argon beam units use a standard generator and grounding pad, but typically use a higher current in coagulation mode to desiccate the target tissues. This property makes it indispensable in hepatic resections.

BIPOLAR (biterminal) is an electrosurgical technique in which the electrosurgical effect takes place between paired electrodes placed across the tissue to be treated (Fig. 5). No patient return electrode is needed. As current passes through the tissue from one electrode to the other, the tissue is desiccated and the resistance increases. As the resistance increases, the current flow decreases. However, bipolar systems also have limitations, as it is more difficult to include a method for electrosurgical vaporization and cutting into the design. Most modern generators provide only a continuous waveform from the bipolar outlets that is identical to the “cut” waveform.

**Figure:**
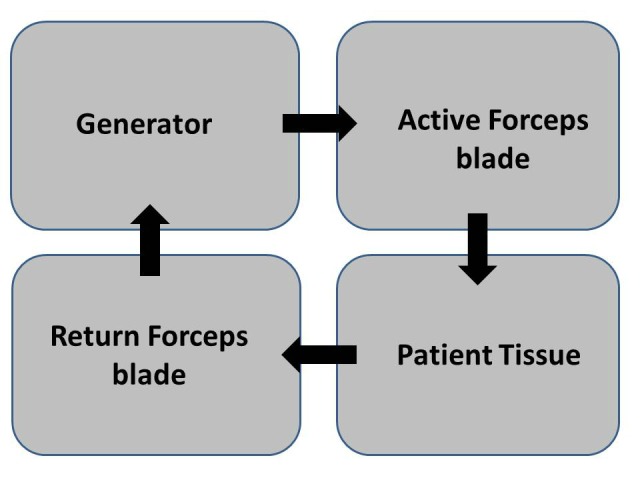
Figure 5: Circuit of a bipolar electrosurgical system

**ADVANCED BIPOLAR INSTRUMENTS**

The addition of computer technology and development of microprocessors that can modulate current density, time and also pressure application over the tissues have revolutionized the electrosurgery and have made them safer and more effective. These systems measure local tissue impedance and/or temperature in an attempt to more accurately define an “end point” for vessel sealing, based on the knowledge that certain temperature thresholds or high impedance levels are associated with complete tissue coagulation and desiccation. Various proprietary bipolar instruments such as Ligasure® (Covidien), PlasmaKinetics ® (Olympus-Gyrus), and EnSeal ® (Ethicon Endosurgery) are available. They enable a surgeon to perform sutureless vessel ligation and sealing of lymphatic channels upto 7mm in size. 

**CONSIDERATIONS IN NEONATAL SURGERY**

It is important for a neonatal surgeon to understand these principles and modify it for optimum desired results with minimal collateral damage. Few important considerations that a neonatal surgeon must understand are discussed below.

- Electrosurgical Generator Power Output- Different generator brands have different output characteristics with varying peak voltages and variable duty cycles at the blend settings. In most instances, the appropriate power output for cutting will be the minimum amount necessary to create the power density at the electrode tip that results in desired combination of vaporization and coagulation. The neonatal surgeon should try to work and set appropriate power levels for the machine and store these data for future use. When more voltage is needed (for example when hemostasis is necessary along an incision line or when transecting tissue that has relatively high impedance), one can use “blended waveforms”, where voltage may be incrementally increased without changing the power settings.
- Low-Voltage Continuous or Modulated (“Cutting”) mode - The low-voltage continuous outputs are generally the most efficient and effective for either cutting (linear vaporization) or for coagulation/ desiccation (both contact and coaptive coagulation). The neonatal surgeon when using monopolar instruments for coaptive coagulation in both open and minimally invasive surgery must select “cut” setting when sealing blood vessels.
- Electrode Surface Area - The power density required for vaporizing or cutting tissue is very high, which in turn requires the use of an electrode with a very small surface area. So with same power and mode of ESU, needle electrode can lead to vaporization whereas wider blade can lead to coagulation. Use of needle electrode should be done in neonatal surgery.
- Use bipolar electrosurgery when appropriate - Bipolar because of its low voltage is safer for neonates in comparison to monopolar. The newer advanced bipolar instruments are excellent for vessel sealing especially in neonatal laparoscopy or thoracoscopy. The open surgery instruments with its capacity to seal 7mm vessels can reduce the use of sutures, which is likely to lead to less adhesion in abdomen [10]. However its use is limited in minimally invasive surgery in neonates by the diameter of delivery probes (smallest is 5mm).
- Tissue Impedance or Resistance - Tissue such as bone, calloused skin, fat, or any previously desiccated tissue will impede passage of current and therefore will inhibit the creation of an electrosurgical effect. In these areas if it is necessary to cut through tissue (for example pelvic osteotomy in bladder exstrophy in neonates), one can increase the voltage by raising the power output or decreasing the duty cycle; both are equally effective.
- Cleaning of the electrode tip is important. As eschar builds up on the tip, impedance increases and can cause arcing, sparking or ignition and flaming of the eschar. When cleaning the electrode, the eschar should be wiped away using a sponge rather than the common scratch pad, because these pads will scratch grooves onto the electrode tip, increasing further eschar build-up
- Compressed tissue volume. The larger the tissue thickness between the jaws of an electrosurgical instrument, regardless whether it is monopolar or bipolar, the greater the number of joules of energy that will be required for complete coagulation and desiccation. What this means is that thicker pedicles will result in more lateral extension of the electrosurgical thermal injury, a factor that may be enhanced with monopolar instruments. So it is advisable that only minimal needed tissue or vessel should be taken between jaws of electrosurgical instruments.
- The patient plate should be properly grounded and should be in contact. If the temperature at the return electrode site increases enough, a patient burn may result. Surface area impedance can be compromised by: excessive hair, adipose tissue, bony prominences, fluids, adhesive failure, scar tissue, and many other variables. Liquids, such as skin preparation solutions, must not be allowed to pool around or leak under the patient plate. These can cause skin burn because the energy flowing towards the neutral electrode can pass through the conducting fluid bridge with a low electrical resistance. This can lead to a high concentration of current density at these points and hence to burning. This is more relevant in neonatal surgery where the operative field and dispersive plate are likely to be adjacent to each other because of smaller patient size.
- Electrosurgical injuries may also affect the surgeon. Surgical gloves are misperceived as offering insulation from radiofrequency current when, in fact, they do not. When using hemostats or a forceps to deliver current to a bleeding vessel from a monopolar electrode (“buzzing the hemostat”), the surgeon should not touch the patient with his free hand. By holding the forceps and then touching the patient with a separate part of the body, a new circuit is created and offers an alternate pathway for current to travel. Instead of through the forceps to the patient, the current in this example travels through the forceps, through the surgeon’s arm and body, and then into the patient. If the surgeon touches “ground”, a new circuit is created and this can lead to injuries.
- The practice of improvisation to insulate the tip of the electrode as is often required in fine operations and while operating on neonates can also prove dangerous (Fig, 6). This practice increases the risk of a fire hazard and is not recommended. Sparks and leakage can and will occur at the junction of the catheter and the insulation on the tip especially when there is a small gap between these two. The high temperature of the electrosurgical active electrode can also melt the catheter. Factory designed insulated electrodes with only exposed tips should be used instead.

**Figure:**
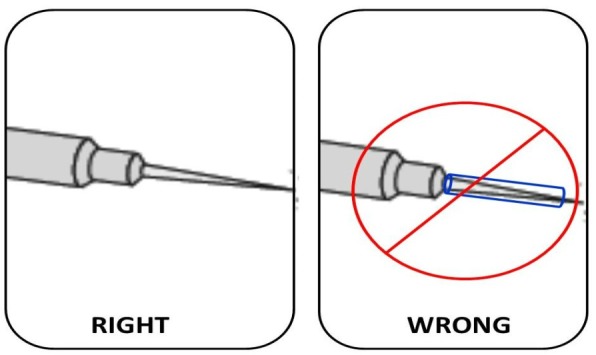
Figure 6: The practice of insulating the tip with make shift tubular insulators should be avoided

**Ultrasonic devices**

An ultrasonic scalpel (US) is a piece of medical equipment used in surgical procedures as an option to a steel scalpel or electro-surgical diathermy. It uses ultrasound technology to cut tissues while simultaneously sealing the edges of the cut. The system typically is composed of a hand-held ultrasonic transducer, generator, hand switch, foot pedal, and scalpel that serve as the cutting instrument. The major manufacturers are Ethicon endo-surgery (Harmonic©), Covidien (Sonicision™ Cordless Ultrasonic Dissection Device), Olympus medical (USG 400), etc.

**How it works**

Ultrasonic dissection instrumentation denatures protein by means of ultrasonic vibrations at a frequency of 55 KHz with a vibratory excursion of 50–100 μm [11]. The two cutting mechanisms of the Ultrasonic dissection instruments are different from that observed with electro surgery or laser surgery. The first mechanism is cavitational cutting and fragmentation. As the blade tip vibrates, it produces large transient pressure changes, which causes cellular water to vaporize at low temperature, rupturing cells, leading to very precise cutting and dissection. The second mechanism for cutting is the actual power cutting offered by a relatively large blade vibrating 55 KHz times per second. The blade edge cuts tissue by stretching it beyond its elastic limit and on a more microscopic level, by breaking molecular bonds. The heat generated from friction of tissue is typically less than 80°C. This minimizes tissue carbonization. 

Advantages over Electrosurgical instruments

The instrument is similar to a surgical diathermy, but advocators suggest it is superior [12]. Ultrasonic surgical devices have been demonstrated to provide excellent hemostasis, efficient transection, minimal lateral thermal damage [13], low smoke generation [14], and no risk of electrical current passage to the patient [15]. These benefits originate from the inherent characteristics of the ultrasonic mechanism in that it can cut through thicker tissue, creates less and safer smoke [16], and may offer greater precision. Tissue damage and wound complications are also reported to be low when compared with electrocautery [17]. Typically, this energy modality is effective for blood vessels between 2mm and 3mm. 

However, the harmonic scalpel is not as easily maneuverable, and takes longer to cut and coagulate tissue. Additionally, while a surgical diathermy can be used to coagulate bleeding tissue at any time, the Harmonic scalpel only coagulates as it cuts.

Considerations in neonatal surgery while using ultrasonic devises

Minimal access surgery in the neonate is an emerging field that has greatly increased its scope in the last decade. Due to space constraints in a neonate use of energy sources that produce less collateral damage and injury to organs due would be preferable. Since electric current does not pass through the neonate, hence it is far safer than electrocautery. Even in open surgeries like taking down vessels in mesentery becomes quite easy and fast with ultrasonic devices. Although no electrical current passes, the instrument tip can become quite hot, leading to chances of direct thermal damage to adjacent tissues in small neonatal area of operation.

## CONCLUSION

Neonatal surgery can be safer and more effective with appropriate use of energy sources. The surgeons should understand the principles of energy sources and utilize these on practice. 

## Footnotes

**Source of Support:** Nil

**Conflict of Interest:** None

